# Pinchy Business: Poland’s Ornamental Crayfish Trade in 2024

**DOI:** 10.3390/ani15172594

**Published:** 2025-09-04

**Authors:** Paweł Wróblewski, Rafał Maciaszek, Wiesław Świderek

**Affiliations:** Department of Animal Genetics and Conservation, Institute of Animal Sciences, Warsaw University of Life Sciences, Ciszewskiego 8, 02-786 Warsaw, Poland

**Keywords:** aquarium, crustacean, invasive alien species, aquaculture, monitoring

## Abstract

The aquarium trade is one of the main pathways for the introduction of non-native freshwater species. Such species include crayfish, which are valued ornamental animals commonly kept in aquaria. Some crayfish have been released into the environment, becoming invasive alien species. Due to the threat they pose to biodiversity and related ecosystem services, they have been subject to legal restrictions as invasive alien species of Union concern. In Poland, examples of species that have entered aquatic ecosystems this way include red swamp crayfish *Procambarus clarkii* and the marbled crayfish *Procambarus virginalis*. Given the highly developed aquarium pet trade in Poland, a detailed analysis of the availability of crayfish in the pet trade was conducted. This study examines the presence and sale of crayfish at locations at zoological trade fairs, shops, and online marketplaces in Poland. Additionally, pricing, the volume of imported crayfish, and their welfare in the year 2024 are presented. Crayfish were recorded in all surveyed locations. In shops and zoological trade fairs, five crayfish species were recorded, including the invasive alien species *P. clarkii* and *C. destructor*. Online advertising platforms featured 15 crayfish species, of which four were invasive alien species. *Cambarellus patcuarensis* was the most commonly sold species in all examined places. Crayfish were often kept in poor condition. Furthermore, many sellers probably disguised the species of crayfish being sold illegally. This work shows that trade in invasive alien crayfish species is still widespread in Poland.

## Simple Summary

Mitigating the threat of the invasive alien species, crayfish, from the aquarium trade is difficult when legal awareness and enforcement are lacking. Despite legal restrictions on some invasive alien species, this study reveals that trade in ornamental crayfish, including invasive species, is still widespread and largely unchecked in Poland. We analyzed the availability, prices, and welfare conditions of ornamental crayfish at zoological trade fairs, in shops, and on online marketplaces in Poland. Crayfish were found at all locations surveyed, with the highest number of offers on online advertisement platforms. The Patzcuaro dwarf crayfish was the most commonly sold species, while red swamp crayfish was the most prominent invasive alien species found across all types of survey locations. We also observed that crayfish were often kept in poor conditions, and many transactions did not include correct species identification, and were likely to circumvent regulations. According to this study, the trade in invasive crayfish is still common in Poland, despite the regulations in place. The presence of these species in the market creates a significant risk of their further introduction into natural ecosystems and the spread of diseases. To address this, more effective oversight, enhanced monitoring of online content, and educational campaigns are urgently needed.

## 1. Introduction

The breeding of ornamental aquatic species is one of the most popular hobbies worldwide [1,2]. This popularity is often attributed to the relatively easy maintenance and attractive coloration of aquarium species. The trade in aquarium animals has been expanding rapidly over the past decade [3]. Some of the largest exporters include Asian countries (e.g., Singapore, Japan, Malaysia, and Thailand), and European countries (e.g., Czech Republic, Poland, and Germany) [4]. Poland is one of the dominant exporters of aquarium animals in the European Union (EU) [5].

The trade in freshwater crayfish within the EU is relatively new but has been gaining popularity [6]. In addition to its economic significance, the pet trade is one of the main pathways for the introduction of non-native species, which may subsequently become invasive alien species (IAS) [7]. Many crayfish available in the trade are carriers of pathogens and fungi that pose a threat to native fauna [8].

Some ornamental crayfish species have already been recorded in natural environments, most likely originating from intentional introductions from aquariums [9]. In Europe, populations of marbled crayfish *Procambarus virginalis* Lyko, 2017 and red swamp crayfish *Procambarus clarkii* (Girard, 1852) have been established in Estonia, Germany, Italy, the Netherlands, and Slovakia [10,11,12]. Populations of these two species have also been found in Poland, including *P. virginalis* in Warsaw and in the nature reserve of Pojezierze Łęczyńskie Landscape Park, also *P. clarkii* in Kraków, Poznań, and Warsaw [13,14,15]. These are not the only aquarium-origin crayfish observed in European aquatic environments. For example, *Cherax destructor* (Clark, 1936) has been found in Italy and Ireland (Bloomer et al., 2024), and *Cherax quadricarinatus* (Von Martens, 1868) has become established in Slovenia [13,16].

Crayfish species available in the Polish pet trade can pose a risk as invasive alien species if released into the environment [17]. Of these, *C. destructor*, *P. clarkii* and *P. virginalis*, are already invasive due to their negative impact on biodiversity and associated ecosystem services. These three species are included in EU Regulation No 1143/2014 on the prevention and management of the introduction and spread of invasive alien species. The directive also aims to ensure proper public awareness by establishing the Central Data Register on Invasive Alien Species, which collects data on their occurrence in the environment and the remedial actions (often expensive) taken against them (General Directorate for Environmental Protection. (n.d.). Central Register of Data on Invasive Alien Species).

We studied the availability of ornamental crayfish in the pet trade in Poland. This analysis is significant because there is an increasing number of illegal introductions of crayfish into the environment [18]. Despite current regulations, the trade in these potentially invasive alien species continues largely unchecked.

## 2. Materials and Methods

A survey was carried out at three zoological trade fairs and in 27 shops. The study took place at the largest zoological trade fairs in Poland, located in Kraków (Balicka 56), Warsaw (ZooEgzotyka), and Łódź (Start Łódź), as well as in shops in these cities. Shops were included if they offered aquarium animals. A year before the study, a review of markets and pet shops was carried out, and on this basis, follow-up examinations were planned for the months of March, June and September 2024. At the same time, online advertisement platforms were also checked, with additional monitoring conducted in December. The following websites were examined: Allegro, Ceneo, Google, Gratka, Facebook Advertising Groups, Facebook Marketplace, and OLX. Advertisement platforms were reviewed once during each chosen month, with searches limited to offers posted in that period (on zoological trade fairs, every single seller offering crayfish is one offer. In turn, one shop is one offer. On advertisement platforms, one seller is one offer). Zoological trade fairs are organized twice a month and were visited by one researcher during each event, while on the same days, another researcher assessed the shops. Based on the collected data, a list of ornamental crayfish available was compiled, and each species was identified according to Kozak et al. [19]. Online searches focused on advertisements for live crayfish specimens. The monitoring process covered Polish-language websites, and advertisement platforms. Crayfish species was searched for directly using the advertisement platform’s search engine with a combination of species and genus names along with keywords such as “for sale,” “price,” “offer,” “buy,” “give away,” and “exchange.” Searches were conducted using both Polish and scientific names, as well as trade synonyms like ‘’Aquarium crayfish, Australian crayfish, blue crayfish, brayfish, CPO, dwarf crayfish, little crayfish, marbled, red crayfish, yabby’’. The collected data included information on the species offered, and additional details such as the region of origin of the advertisement, the type of online platform, the price, and potential methods used to conceal illegal sales.

The method of keeping crayfish at zoological trade fairs was also assessed. Based on Elwood [20], it was determined whether the method of keeping the animals affected their welfare. The welfare of crayfish was assessed by the individual ability of the crayfish to move in the container.

All available crayfish species were recorded, noting the common and scientific names used, the number of specimens available, the type of seller, and the price. The price for a crayfish in most advertisements referred to a single specimen. However, some offers provided the price per kilogram of crayfish. In such cases, the average price per specimen was calculated based on its average mass using the formula: Cr = p·m [19]. Cr—price of one crayfish (in USD), m—mass of one crayfish (in kg), p—price per 1 kg of crayfish (in USD). The currency was 1 USD = 3.74 PLN. Then, having the average price of a crayfish from a given offer, the average price of all crayfish was calculated.

The obtained data were subjected to statistical analysis. Nonparametric methods were used because the variables did not meet the requirements of normal distribution (Shapiro–Wilk test) and homogeneity of variances (Levene’s test). The Kruskal–Wallis test was used to assess differences between places offering crayfish sales (zoological trade fairs, shops, advertisement platforms) in different study periods (March, June, September, December). The relationship between the price and the availability of crayfish was assessed using the Spearman rank correlation. All statistical calculations were performed using Statistica 13.1.

## 3. Results

A total of 134 offers featuring crayfish were recorded. The largest number of crayfish (1990) was offered on advertisement platforms, significantly (*p* < 0.05) fewer at zoological trade fairs (553 crayfish) and in shops (126 crayfish) (Table 1). The greatest diversity of species was recorded on advertisement platforms (15), while only five were offered at zoological trade fairs and in shops.

The most frequently offered species across all surveyed locations was the Patzcuaro dwarf crayfish *Cambarellus patzcuarensis* (Villalobos, 1943) in an orange morph, which is not the normal wild phenotype. The least frequently offered species (one) included *Cherax alyciae* (Lukhaup, Eprilurahman and von Rintelen, 2018), *Cherax bosemani* (Lukhaup and Pekny, 2008), *Cherax snowden* (Lukhaup, Panteleit and Schrimpf, 2015). Among invasive alien species of Union concern, *P. clarkii* had the highest number of offers across zoological trade fairs, shops, and advertisement platforms, while signal crayfish *Pacifastacus leniusculus* (Dana, 1852) which is not an aquarium species, had the lowest.

Among the 15 crayfish species, the lowest average price per specimen was recorded for *P. virginalis*, while the highest price per specimen was observed for *C. alyciae* (Table 1). The correlation between the price and the availability of crayfish is (r = −0.58).

**Figure 1:**
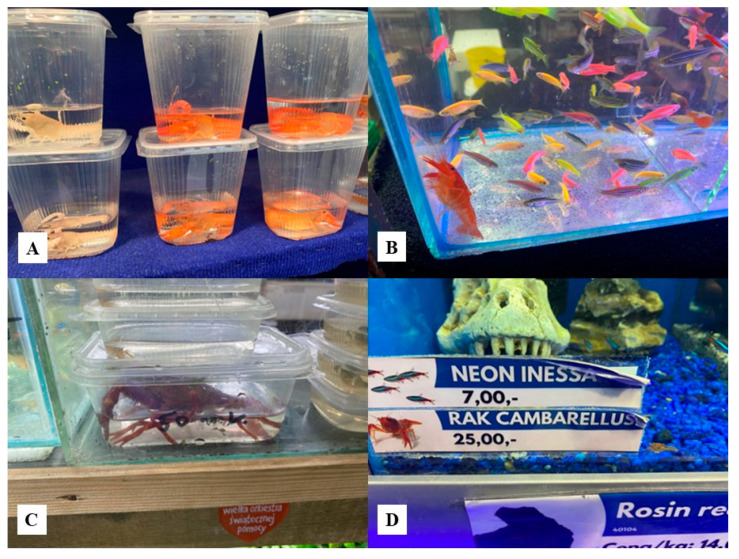
Some invasive *P. clarkii* presented in zoological trade fairs (**A**–**C**), and non-invasive. *C. texanus* (**D**) presented in a shop (P. Wróblewski).

The crayfish were kept in containers that were too small to allow the crayfish to move freely (Figure 1A,C). This method of storing crayfish was observed at every seller at zoological trade fairs.

A comparison of trade and scientific names for crayfish is presented in Table 2.

The highest percentage share in the trade of all crayfish species on classified advertisement platforms was recorded in the Silesia Voivodeship (19.3%), the Lower Silesian Voivodeship (18.2%) and the Pomeranian Voivodeship (16.0%) (Table 3). There were no offers noted in the Opole, Lubusz and Warmian–Masurian Voivodeships. The highest percentage share in the trade of invasive non-native crayfish species was recorded in Silesia Voivodeship (33.1%), and Podlaskie (12.4%) Lower Silesia (10.2%). No offers were recorded in Opole, Lubusz, Lesser Poland, Subcarpathian, Świętokrzyskie, and Warmian–Masurian Voivodeships.

## 4. Discussion

The present study provides an analysis of the zoological market for crayfish. To our knowledge, this is the first publication providing a compilation of crayfish species present in the pet trade in Poland, including zoological fairs, advertisements, and pet stores.

The high availability of crayfish in stores suggests that they are significant in the ornamental animal trade in Poland. Furthermore, their presence was noted in all pet trade fairs surveyed, further underscoring the importance of crayfish in the trade. Advertisement platforms accounted for the majority of offers across all species. Several species, such as *C. texanus* and *P. virginalis*, were absent from zoological trade fairs, indicating a potential limitation in species diversity at such events. In contrast, *C. patzcuarensis* was consistently present across all surveyed locations and was the most frequently listed species overall. Notably, *P. leniusculus* (designated as an invasive alien species in the EU) and *C. snowden* (not designated as an invasive alien species) appeared on advertisement platforms, further underscoring the role of digital marketplaces in the distribution of both native and non-native species. This disparity suggests that advertisement platforms may serve as a more comprehensive reflection of market availability and demand.

The increasing popularity of crayfish among aquarists in Poland, along with the country’s status as one of the leading importers of aquarium animals, has also contributed to the development of this study [5,21,22]. Analyses from southern neighboring countries reveal a widespread occurrence of invasive crayfish species in the aquarium industry (Patoka et al., 2015) [17]. Moreover, numerous studies highlight the potential negative impact on native crayfish populations, as aquarium crayfish species may serve as vectors for crayfish plague *Aphanomyces astaci* (Schikora, 1906) and white spot syndrome [17,23].

The most popular aquarium crayfish in Poland is *C. patzcuarensis*, a small crayfish that is easy to keep in small aquariums [24]. The second most popular species, *Cambarellus diminutus* (Hobbs, 1945), is also small, supporting that size matters for hobbyists buying crayfish. They can be kept with some fishes in the same aquarium with neither harming the other, which is not possible with other crayfish species [25,26]. This may explain their exceptionally high popularity. The fewest listings were recorded for *C. alyciae*, *C. bosemani*, and *C. snowden*. Their low popularity may be due to the fact that these species were only recently discovered, and their descriptions are still relatively unknown. These crayfish grow to larger sizes than *C. diminutus* or *C. patcuarensis*. *Cherax* genus range in size from 100 to 150 mm in total length [27]. One might expect large species to be less available. However, they may become very popular in the future due to their attractive color.

Advertising platforms offer the widest range of species available for sale, which may result in a faster sale than, for example, at zoological trade fairs [28]. *Cambarellus patzcuarensis* is strongly present in shops, zoological trade fairs, and online stores. It is the species with the widest reach and the highest number of listings. Among invasive alien species, *P. clarkii* dominates both in terms of the number of listings and reach across various sales channels. Among zoological trade fairs, shops, and classified advertisement platforms, it was the latter that featured the most offers for this species. What may be surprising is that *P. clarkii* is sold in aquarium stores. This could be due to a lack of oversight on advertising platforms and in shops, as well as a general lack of awareness among sellers. In the case of aquarium shops, sellers probably intentionally offer invasive alien species because they can be sold better than other crayfish species, because of their low price.

The results obtained clearly indicate a significant price differentiation among the various species of ornamental crayfish available on the market. The lowest average unit price was recorded for *P. virginalis* (1.07 USD), while the highest was noted for *C. alyciae* (32.07 USD). Size might be a factor, as large animals might be expected to command larger prices. This considerable price disparity may be attributed to several factors, including the challenges associated with breeding, visual appeal, the market availability of a given species, and demand among hobbyists. The data show that more expensive crayfish species are generally less readily available, and this correlation is moderate (r = −0.58). This means that, on average, as the price increases, the number of places where they can be found decreases. The cheapest species, such as *C. patzcuarensis*, are relatively widely available, while the most expensive species, such as *C. snowden*, appear sporadically. This suggests that the price in the crayfish market partly reflects their rarity.

The invasive alien species *P. virginalis* exhibits parthenogenetic reproduction, which makes it exceptionally easy to breed under aquarium conditions. This species is among the most frequently imported ornamental crayfish in Central Europe, contributing to its high availability and relatively low unit price [29,30]. Furthermore, due to its ease of cultivation and minimal environmental requirements, *P. virginalis* has become a popular choice among novice aquarists.

In contrast, the high prices of species from the *Cherax* genus, particularly *C. alyciae*, may result from limited availability, transportation challenges, and strong interest from collectors. These species are characterized by vivid, attractive coloration and larger body size, making them more desirable. The import volume of *Cherax* species into Europe, including the Czech Republic and Poland, is noticeably lower than that of species from the *Procambarus* or *Cambarellus* genera, further enhancing their market value [31].

The advertisement platforms study shows that trade names for crayfish are often ambiguous, as a single commercial term may refer to multiple different species, while a single species can appear under various names [32]. In particular, *C. patzcuarensis* is marketed as “CPO,” “dwarf crayfish,” “little crayfish,” or “red crayfish,” which highlights its considerable prominence in the ornamental trade. Such inconsistencies complicate monitoring efforts and may lead to misidentifications, especially when both non-invasive aquarium species and highly invasive taxa are sold under the same designation [26]. This underscores the necessity of standardizing nomenclature and confirms the special appeal of dwarf crayfish as an attractive species in the aquarium market [33].

The study revealed a distinct geographical variation in crayfish trade via classified advertisement platforms across Poland. Notably, the Silesian Voivodeship stood out in terms of the trade in both all crayfish species and invasive alien species, which may indicate an intense level of aquarium-related and commercial activity in this region. Significant shares were also recorded in the Lower Silesian and Pomeranian Voivodeships, likely associated with high population densities and a well-developed online trade infrastructure. Conversely, voivodeships such as Opole, Lubusz, and Warmian–Masurian exhibited either no or very low levels of crayfish trade. This could reflect a lower interest in aquarium keeping, a smaller user base of online classified platforms, or a higher degree of environmental awareness.

Of particular concern are the data related to the trade in invasive alien species, which pose a serious threat to native populations [11,34]. The presence of invasive alien species of Union concern in the market may facilitate their further introduction into natural ecosystems, increasing the risk of native species displacement and the spread of pathogens such as *A. astaci*, the causative agent of crayfish plague. There is a real risk of releasing the invasive alien species detected in the study. As in the Netherlands, commercially available crayfish could be potential invasive alien species in Europe [35].

As noted [11], the global trade in crayfish as pets often occurs in the absence of adequate regulation, creating the potential for uncontrolled dissemination of invasive alien species. Our findings confirm that Poland is no exception, and this issue may intensify in the context of the growing popularity of aquarium hobbies. Moreover, [36] this study highlights Eastern Europe as a region that is particularly vulnerable to the influx of alien species. Given its extensive freshwater systems and thriving online trade, Poland may act as a gateway for the further spread of invasive crayfish species across the region [14]. Invasive alien species of aquarium-origin crayfish are being caught [37].

Some practices are likely meant to circumvent legal regulations governing the trade of alien and invasive alien species, as well as avoiding detection by moderators of sales platforms. The observed strategies include, among others, shortening or altering Latin and common names, the use of pseudonyms (e.g., “mar-murkowy”), or the complete omission of species identification while simultaneously providing characteristic images [7,38].

Similar tactics have been described in the context of the illegal trade of invasive plants in Australia [39], where it has been shown that such trade often relies on seemingly innocuous keywords or hidden species names embedded within the content of advertisements. These techniques allow interested buyers to locate listings without raising suspicion among platform moderators or regulatory authorities. Likewise, the Salamandra report (2023) [28] highlights that in Poland, cases of selling invasive alien species via e-commerce platforms are also widespread, frequently occurring without the required permits and often involving attempts to conceal species identity from oversight mechanisms.

The highest number of listings recorded on classified advertisement platforms may suggest that this method of selling crayfish is the most popular. This could be due to the ease of posting an ad and the potential for quick profit. Zoological trade fairs, on the other hand, require an entry fee and travel to the location, which may contribute to their lower popularity among crayfish sellers. In the context of the trade of ornamental crayfish at zoological trade fairs in Poland, it is relevant to refer to the findings of a study on the welfare of exotic animals presented at such events. Although the research conducted [40] focused on reptiles and amphibians, its conclusions may be analogously applied to ornamental crayfish. The study revealed that many crayfish were kept in containers that were too small for them, lacked proper substrate, and failed to provide environmental enrichment. Abnormal postures and stress-related behaviors were also observed, indicating poor welfare and discomfort among the animals. Despite existing legal regulations, violations of basic welfare standards were commonly recorded. Similarly, ornamental crayfish displayed at fairs are often subjected to comparable issues. They are frequently housed in overly small containers, without appropriate substrate or hiding places, which can result in stress, increased aggression, and overall diminished welfare [41]. Inadequate holding conditions may also elevate the risk of disease and mortality among the animals [42]. Therefore, there is a clear need for the introduction and enforcement of more rigorous welfare standards for crayfish displayed at pet fairs. It is recommended that organizers and sellers ensure sufficiently large containers, appropriate substrates, and shelters, and provide proper husbandry information to potential buyers. The necessity of investigating crayfish from the pet trade is evident, as they may pose risks to native species [43] and act as carriers of pathogens detrimental to native populations [44,45].

## 5. Conclusions

The study found that the trade in ornamental crayfish remains widespread in Poland, despite legal restrictions on invasive alien species. Crayfish were found in all surveyed locations, with the greatest diversity and quantity of sales offers observed on advertisement platforms. The presence of invasive species such as *P. clarkii* and *P. virginalis*, combined with inadequate labeling and inadequate welfare conditions, highlights serious gaps in law enforcement and awareness. These findings underscore the urgent need for more effective monitoring, automated detection of illegal online offers, and targeted educational campaigns to reduce ecological risks.

## Figures and Tables

**Table 1:** Average price and number of freshwater crayfish species offered at zoological trade fairs, shops and advertisement platforms.

Species	Price (USD)	Zoological Trade Fairs	Shops	Ad Platforms	Total Availability
*Cambarellus diminutus*	4.09	146	14	1268	1428
*Cambarellus patzcuarensis*	5.21	197	61	258	516
*Cherax quadricarinatus*	10.53	60	18	100	178
*Cambarellus texanus*	3.53	-	-	139	139
*Procambarus clarkii* (IAS EU)	11.38	63	30	19	112
*Astacus astacus*	2.67	-	-	110	110
*Cherax destructor* (IAS EU)	23.27	67	3	5	75
*Pontastacus leptodactylus*	5.83	-	-	36	36
*Cherax holthuisi*	25.14	-	-	31	31
*Procambarus virginalis* (IAS EU)	1.07	-	-	17	17
*Cherax pulcher*	25.39	-	-	3	3
*Cherax bosemani*	24.05	-	-	1	1
*Cherax snowden*	26.72	-	-	1	1
*Pacifastacus leniusculus* (IAS EU)	6.68	-	-	1	1
*Cherax alyciae*	32.07	-	-	1	1
Total		533 *	126 *	1990 *	2649
March		207	37	238	482
June		156	35	389	580
September		170	54	185	409
December		-	-	1178	1178

* significant differences—(*p* < 0.05), correlation (Price USD—Total availability): r = −0.58, *p* < 0.05.

**Table 2:** Trade names of crayfish frequently used on advertisement platforms, along with their scientific names.

Trade Name	Scientific Name
aquarium crayfish (rak akwariowy)	*C. patzcuarensis*, *C.diminutus*, *C. alyciae*
Australian crayfish (rak australijski)	*C. destructor*, *C. quadricarinatus*, *C. bosemani*
blue crayfish (rak niebieski)	*P. alleni*, *C. pulcher*
CPO (rak karłowaty pomarańczowy)	*C. patzcuarensis*
crayfish (rak)	*P. leniusculus*, *P. leptodactylus*, *C. holthuisi*, *A. astacus*
dwarf crayfish (rak karłowaty)	*C. patzcuarensis*, *C. diminutus*, *C. texanus*
little crayfish (mały rak)	*C. patzcuarensis*, *C. diminutus*
marbled crayfish (rak marmurkowy)	*P. virginalis*
red crayfish (rak czerwony)	*C. patzcuarensis*, *P. clarkii*
yabby (raki rodzaju *Cherax*)	*C. destructor*, *C. snowden*

**Table 3:** Offers of sale of alien species of crayfish on ad advertising platforms, divided by voivodeships and calculated by the number of inhabitants.

Shorten Name of Voivodeship	A (%) *	B (%) *
Lower Silesian Voivodeship	18.2	10.2
Kuyavian-Pomeranian Voivodeship	7.5	7.0
Lublin Voivodeship	8.8	6.9
Lubusz Voivodeship	0.0	0.0
Łódź Voivodeship	3.8	5.9
Lesser Poland Voivodeship	3.9	0.0
Masovian Voivodeship	5.8	5.5
Opole Voivodeship	0.0	0.0
Subcarpathian Voivodeship	1.5	0.0
Podlaskie Voivodeship	5.4	12.4
Pomeranian Voivodeship	16.0	6.3
Silesian Voivodeship	19.3	33.1
Świętokrzyskie Voivodeship	2.6	0.0
Warmian–Masurian Voivodeship	0.0	0.0
Greater Poland Voivodeship	1.8	4.1
West Pomeranian Voivodeship	5.5	8.6

* A—offers of all crayfish species, * B—offers of invasive alien crayfish species.

## Data Availability

The data presented in this study are available on request from the corresponding authors.
